# The Re-/Up-Cycling of Wood Waste in Wood–Polymer Composites (WPCs) for Common Applications

**DOI:** 10.3390/polym15163467

**Published:** 2023-08-19

**Authors:** Carmen-Alice Teacă, Asim Shahzad, Ioana A. Duceac, Fulga Tanasă

**Affiliations:** 1 Center of Advanced Research in Bionanoconjugates and Biopolymers, “Petru Poni” Institute of Macromolecular Chemistry, 41A Grigore-Ghica Vodă Alley, 700487 Iaşi, Romania; cateaca14@yahoo.com; 2 College of Aeronautical Engineering, National University of Sciences and Technology, Risalpur 23200, Pakistan; asim.shahzad@cae.nust.edu.pk; 3 Polyaddition and Photochemistry Department, “Petru Poni” Institute of Macromolecular Chemistry, 41A Grigore-Ghica Vodă Alley, 700487 Iaşi, Romania; duceac.ioana@icmpp.ro

**Keywords:** wood waste, wood–polymer composites, recycling, up-cycling, compatibilization, nanofillers, wood–PP composites, wood-plasticized starch composites, properties

## Abstract

Wood–polymer composites (WPCs) are a class of materials intensively studied and promoted in the context of sustainable development, mainly when aspects related to the increasing awareness of environmental issues and waste management are considered. Feasible opportunities for producing WPCs with value-added properties intended for common applications emerge when polymers, either synthetic or from renewable resources, raw or waste, are employed in re-/up-cycling approaches. In this context, some examples of easily achievable WPCs are presented herein, namely, formulations based on different wood waste and polymer matrices (synthetic: polypropylene and malleated polypropylene as a compatibilizer; natural: plasticized starch). Their level of performance was assessed through different characterization methods (FTIR, WAXD, TGA, DSC, mechanical test, etc.). The benefits and limitations of this approach are also discussed.

## 1. Introduction

**Figure 1:**
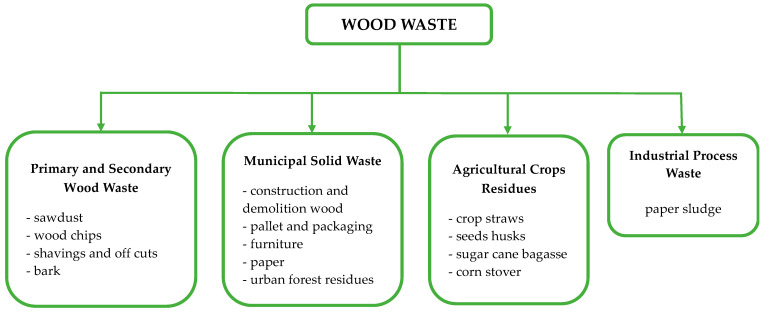
Various categories of wood waste (re-drawn from [19]).

Wood consumption is increasing throughout the world, and it is estimated that in Europe, the demand will exceed the supply by 2030 [1]. This consumption rate yields a huge amount of wood waste of various types, as presented in Figure 1. The main source of post-consumer wood waste is municipal solid waste (MSW), which consists of damaged furniture and miscellaneous durable goods (e.g., cabinets for electronic equipment), packaging (crates, pallets), construction materials (decking, fencing, windowsills, and so on), etc. It is, therefore, of paramount importance to recover and re- and up-cycle wood waste as much as possible. Moreover, only a third of the wood waste is reused in Europe (33%, mainly particle boards), while most wood waste is discarded by landfill and incineration, and energy recovery (37 and 30%, respectively) [2].

As the European Union (EU) is moving towards a circular economy, as highlighted in the circular economy action plan [2], waste recycling is considered one of the main driving forces when it comes to significantly minimizing the consumption of virgin materials and, subsequently, increasing the amounts of waste recycled from 50% of plastic and 25% of wood waste by 2025 up to 55% for plastic and 30% for wood waste by 2030 [3].

Wood biomass is one of the most important natural resources used in different applications, but it comes with significant wood waste generation in the final part of wood products’ life cycles. Wood waste recovery makes the replacement and conservation of primary resources possible through different strategies [4], including re- and up-cycling approaches. In addition, raising public global awareness has become one of the driving forces in the wood waste management issues [5]. In this context, the prevailing trend is to design and produce new materials according to circular economy principles so that the recovery and recycling of wood waste yield better results than the energy recovery strategy.

The most important wastes resulting from wood processing that are further employed for the manufacture of wood–polymer composites (WPCs) are sawdust, flour, shavings, and bark [6,7,8,9], even though all these by-products are still disposed of through incineration and landfill in high amounts [10]. A recent trend is to valorize waste materials through cyclical reutilization as composites (for example, reprocessed WPCs waste [11]) and recycled wood fibers from sawdust resulting from the processing of phenolic resin wood products [12].

Pine needles represent another important part of biomass waste, typically generated by *Pinus* softwood species, which are used in WPCs as reinforcement (as particles or fibers) for various polymer matrices (e.g., polypropylene, phenol–formaldehyde, urea–formaldehyde, isocyanate, resorcinol–formaldehyde) [13,14,15,16,17], yielding materials with interesting mechanical, thermal, and thermoacoustic properties, as well as flammability and biological resistance.

In the case of wood fibers, they are generally obtained through refining or pulping [18] when fibers of various sizes are obtained and further used for WPCs with specific properties.

Wood as reinforcement in WPCs, provided from either virgin or recycled materials, ensures several advantages, such as being low cost, having a wood-like appearance, and being environmentally friendly, as well as renewability and recyclability [7,20]. Plastic processability enables WPCs to be used in a wider range of applications and with increased economic value, but the cost-effectiveness of using recycled waste as WPC components has to be assessed specifically for each case [21]. Given its intrinsic low biologic resistance, wood waste undergoes degradation through biochemical reactions, which can influence the performance of WPCs in terms of mechanical and thermal properties when employed in compatibilized formulations that may also include various additives [22,23,24,25,26]. At the same time, WPCs with biomass waste as reinforcement can achieve particular valuable properties, such as thermal and acoustic insulation [27]; hyperaccumulating capacity for wastewater decontamination (e.g., *Pteris vittata* is used to retain high amounts of arsenic from polluted water [28]); enhanced recyclability [29]; higher porosity [30]; and superior mechanical properties [31,32,33].

Three main issues must be addressed when dealing with WPCs, namely, compatibilization of the respective components, upgrading the WPCs properties, and the environmental impact (life cycle assessment, LCA). The challenges for each issue will be briefly outlined below.

First of all, in order to enhance the interfacial properties between wood filler and polymer matrix, given the hydrophilic nature of wood particles, pre-processing treatments are employed. The main approaches refer to (1) the surface modification techniques applied to wood—chemical and biochemical (enzyme), physical, radiative, and mixed methods [34]. (2) The use of compatibilizers incorporated into the polymer matrix—such as malleated polyolefins, which are highly recommended for the respective polyolefin matrices [22]; ethylene diamine tetraacetic acid (EDTA), a reactive compatibilizer for poly(lactic acid)/poly(butylene adipate-co-terephthalate) mixed matrix [35]; ionic liquids [36]; glycidyl methacrylate and polyethylene-co-glycidyl methacrylate [37]; and mixtures of malleated polyolefins [38]. And lastly, (3) any combination of methods wisely chosen and applied simultaneously to both matrix and reinforcement.

Experimental data reported in the literature concluded that all these procedures remarkably improved the compatibility between components with beneficial effects on the final properties of WPCs.

Secondly, a significant amount of research has been carried out in the last decade to study the possibilities of using nanomaterials to upgrade the properties of WPCs made of wood waste [39]. Nanostructured materials are being studied in this context from two points of view: (1) as an additive for the polymer matrix and (2) as potential modifiers of wood by deep penetration into the wood cell wall/tracheids.

**Figure 2:**
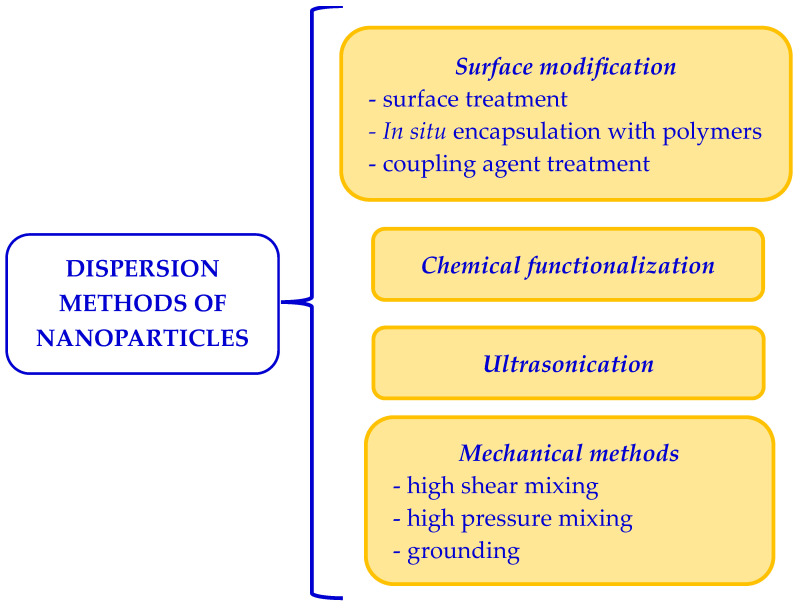
Different techniques used for dispersing nanomaterials (re-drawn from ref. [39]).

Due to their high surface energy, nanoparticles tend to agglomerate upon mixing with the polymer matrix. At the same time, depending on the processing procedure, device, and parameters, a secondary agglomeration may also occur. This is mainly attributed to the functional groups on the surface of nanoparticles that play a critical role in the compatibility between nanoparticles and the immediate environment [40]. Therefore, different methods and techniques were used to obtain a homogeneous and stable dispersion of the nanoparticles inside the matrix but also to allow the uniform penetration of the nanoparticles into the wood tracheids (Figure 2).

As concerns the penetration of nanoparticles into the wood tracheids, this phenomenon strongly depends on the wood structure [39]. The nanoparticles used as modifiers can easily penetrate wood through cell wall pores, where they can bond with the available –OH groups in cell wall constituents, which is a real benefit. However, the penetration of nanoparticles can cause a significant reduction in pore size, physically blocking the free movement of small mobile molecules (i.e., water molecules) through cell wall pores, which is a drawback.

Various nanoparticles have been used to manufacture WPCs with improved properties such as dimensional stability, water resistance, flexural strength, hardness, compression strength, fire retardancy, and thermal stability. Thus, it was reported that graphene nanoplatelets afforded WPCs with significantly increased electric and thermal conductivity [41,42]; nanoparticulate clays enhanced the mechanical, chemical, and thermal properties of composites [43]; colloidal silica nanoparticles afforded WPCs with higher density and dimensional stability [44]; a combination of cellulose nanofibers and clay nanoparticles caused a spectacular increase in mechanical properties of the corresponding composites [45]; and Cu and MgO nanoparticles granted antifungal properties [46,47].

Thirdly, according to the International Organization for Standardization (ISO), life cycle assessment (LCA) is one of the environmental management techniques that “addresses the environmental aspects and potential environmental impacts throughout a product’s life cycle from raw material acquisition through production, use, end-of-life treatment, recycling, and final disposal” (EN ISO 14040:2006 [48]; EN ISO 14044:2006 [49]). LCA proved to be an extremely competent tool for assessing possible implications and/or mechanisms when the environmental implications of different resources were evaluated, as was conducted in the case of WPCs [50].

Results of the previous investigations of WPCs’ LCA can be broken down into two categories: (i) studies comparing WPCs to components (e.g., wood) and (ii) studies evaluating the environmental consequences of WPCs manufactured from different raw materials (virgin versus recovered materials).

Studies on WPCs made of virgin wood, as well as waste wood, and both containing HDPE, as well as recycled HDPE [51,52], indicated that composites made entirely of waste had a lower environmental impact because the production of virgin polymers is a highly energy-consuming process. Nevertheless, further analysis is required in order to precisely assess the input of every stage of the composite’s life cycle.

Following this, some experimental results regarding WPCs made of both a synthetic polymer matrix are presented, namely polypropylene PP and malleated PP as a compatibilizer, and a natural polymer matrix, glycerol-plasticized starch, using different types of wood waste (pine wood sawdust, fir tree needles, and beech wood sawdust) and appropriately selected additives. Some of the most relevant properties of these composites are also assessed and discussed.

## 2. Materials and Methods

### 2.1. Compatibilized Wood–Polymer Composites

#### 2.1.1. Materials

Polypropylene (PP, Malen P F401), provided by Basell-Orlen as pellets, has been used as a matrix. PP characteristics: MFI 3.2 g/10 min (230 °C, 2160 g), density 0.9 g/cm^3^. Malleated polypropylene (MAPP, Fusabond MZ 203D) having a molecular weight of 40,000 g/mol, MFI 102 g/10 min (190 °C, 2160 g), and MA content 0.74 wt%, which was courteously supplied by DuPont Company, Wilmington, Delaware, USA has been chosen as compatibilizer and was used as received. The organically modified clay employed in the composite formulations was Cloisite^®^ 20 A (Cl20A, from Southern Clay Products, Inc., Austin, Texas, Statele Unite), which is dimethyl bis(hydrogenated tallow) quaternary ammonium chloride montmorillonite (hydrogenated tallow is a blend of saturated alkyl chains and contains ~65% C18, ~30% C16 and ~5% C14). Clay characteristics: organic load 95 mequiv/100 g clay; organic content 39.6 wt%; density 2.83 g/cm^3^, d_001_ = 24.2 Å by XRS; particle size distribution: <15 μm (90%); moisture < 2%. The selected reinforcement (wood waste of *Pinus radiata*) was collected as sawdust from a local furniture manufacturer. The chip sizes varied from 0.1 to 7 mm, as determined by using vibrosieves with size fractions of 0.2–0.5, 0.5–1, 1–2, 2–3, 3–5, and 5–7 mm (hereafter, the fraction size is defined as the upper limit of the interval). Each fraction was weighed with a microbalance. Prior to WPCs processing, wood particles were dried to 1–2% moisture content using a vacuum oven at a temperature of 80 °C. The wood received no pre-treatment before processing.

#### 2.1.2. WPCs Processing

*Melt mixing.* The polymer pellets, compatibilizer, and clay were dried in a vacuum oven at 70 °C for 24 h and then fed to a HAAKE RHEOCORD 9000 mixer (equipped with two internal roller mixers and a mixing chamber with a capacity of 50 cm^3^). The processing temperature was set at 190 °C to prevent wood degradation by thermally initiated biochemical reactions, and the screw speed varied between 100 and 150 rpm.

The processing took place in two stages: (1) first, PP, MAPP, and Cl20A were blended in order to obtain compatibilized hybrid materials, and then (2) hybrids were compounded with wood chips at 190 °C. Finally, composites were allowed to cool in the open air and then pelletized. All components were calculated as parts per 100 parts resin (phr). In Table 1, all composite formulations and the corresponding sample codes are given.

#### 2.1.3. Characterization

The weight of wood particles was determined with a Sartorius 4431 microbalance (Gottingen, Germany). Ten particles of each fraction were selected for measurement.

For the thermogravimetric analysis (TGA), a thermogravimetric analyzer (Paulik−Paulik−Erdey-type Derivatograph, MOM—Budapest, Hungary) was used. Each specimen was heated from room temperature up to 600 °C at a heating rate of 12 °C/min. The weight of each sample was 50 mg, and the heating unit was continuously flushed by an air flow (30 mL/min) during testing.

Differential scanning calorimetric (DSC) thermograms were registered using a Pyris Diamond DSC (Perkin Elmer, Waltham, Massachusetts, USA) at a heating rate of 10°C/min and under dry N_2_ atmosphere. The samples were heated up to 200 °C. The melting temperature (*T_m_*), enthalpy (Δ*H_m_*), crystallization temperature (*T_c_*), crystalline enthalpy (−Δ*H_m_*), and crystallinity (*X_c_*) were determined after the melt–crystallization process. The heat of fusion of the fully crystalline PP has been considered a reference, with calculations of crystallinity resulting in 198 J/g [53,54].

The mechanical properties of composite samples (dimensions 150 × 10 × 5 mm), namely tensile strength and modulus, were determined according to ASTM D 638–01 and ASTM D790–00 specifications, using a mechanical tensile machine (FU-1000; Rauenstein, Germany) with a crosshead speed of 20 mm/min. All samples were conditioned at room temperature (23 ± 2 °C) and 50 ± 5% relative humidity for at least 40 h before testing. At least five samples were tested for each type of measurement.

### 2.2. Wood–Plastic Composites with Starch Matrix

#### 2.2.1. Materials

Starch (S) derived from corn was provided by a local commercial store. Glycerol, employed as plasticizer, was provided by Fluka (98% purity, Fluka Chemical, Darmstadt, Germany). The wood waste, previously ground to obtain particles by using a Retsch PM 200 planetary ball mill and employed as fillers, includes fir tree needles (FNW) and beech wood sawdust (BSW), which were provided by local sources. Particles passed through a 0.40 mm sieve were employed for preparation of composites. The fir tree needles (having 300–350 mm length) were collected from a Christmas tree, being previously stored at room temperature for a period of 12 months (moisture content around 10%). The beech wood sawdust was obtained under industrial conditions from a local sawmill (moisture content reached around 5% after oven-drying). The wood received no pre-treatment before processing.

#### 2.2.2. Methods

##### Method Employed for Production of Wood Waste Composites with Starch Matrix

Plasticized starch as polymer matrix for composites with waste biomass was considered due to its advantages, such as easy and economical blending possibility, biodegradability, and flexibility in adjusting its properties to the needs of a specific application. The mixture constituted by starch (5 g), distilled water (100 mL), and glycerol (1 mL) was heated under continuous stirring at 90 °C for 30 min for the complete plasticization of starch. This polymer matrix was employed for production of composites using different wood waste amounts ranging from 10% up to 30% (coded as CS-FNW and CS-BSW); a reference sample was also considered, comprising only glycerol-plasticized starch (coded as CS). The doctor blade technique (a fast-coating approach) was employed for the production of composites. This method relies on using a blade with a slit width of 0.8 mm which allows the polymer mixture to easily fall in drops and spread on glass plates when composite films are formed. These glass plates covered with polymer films were further maintained in a vacuum oven (at 50 °C for 24 h) for degassing and up to a constant weight. A digital micrometer was employed for the determination of the films’ thickness when an average value of 0.2 mm was evidenced. Afterwards, the films were subjected to an air-cooling process and detached from the glass surfaces to be investigated. These samples, whose codes are given in Table 2, had around 9 wt% water content after their preconditioning in a climate chamber (at 25 °C and 50% RH) for at least 48 h prior to the analysis.

##### Methods Employed for Characterization of Wood Waste Composites with Starch Matrix

FTIR Spectroscopy Investigation

A Bruker Vertex 70 spectrophotometer was employed for acquiring FTIR spectra for the plasticized corn starch film and composite samples with wood waste, using a 4 cm^−1^ spectral resolution and a 400 up to 4000 cm^−1^ scanning range.

WAXD Analysis

The crystalline structure of starch and glycerol-plasticized starch was evidenced by using a Bruker AD8 ADVANCE X-ray diffractometer (BRUKER AXS Advanced W-ray Solutions GmbH, Karlsruhe, Germany) (Cu Kα radiation at 60 kV and 50 mA at room temperature). The diffraction angle 2θ ranged from 10 to 30° at a rate of 2° min^−1^ for detection of the scattered radiation.

Water Absorption Measurements

Rectangular film strips from composite samples, with dimensions of 10 mm × 10 mm × 0.2 mm, previously vacuum-dried at 90 °C overnight, were employed for the water absorption measurements. The molecular diffusion can be considered one-dimensional as these film specimens are supposed to be thin enough. After measuring their weight, film specimens were conditioned at 25 °C and a relative humidity (RH) of 95% in a desiccator containing sodium sulfate. Further, samples were removed at specific time intervals and gently blotted with tissue paper in order to remove the excess water on the surface. Water absorption values were calculated with Equation (1) as follows:

water absorption (%) = [(Wt − W_0_)/W_0_] × 100 (1)

where Wt and W_0_ represent the specimen weight at time t and before exposure to 95% RH, respectively. The determinations were performed in triplicate.

Optical Properties Measurements

A JENWAY 6405 UV–VIS spectrophotometer (Cole-Parmer, St. Neots, UK) was employed for evaluation of the optical properties of the films, as opacity which is defined as the area under the absorbance spectrum between 400 and 800 nm, according to the ASTM D 1003-00 method (ASTM D 1003-00 Standard Test Method for Haze and Luminous Transmittance of Transparent Plastics [55]). Rectangular pieces (1 × 2.5 cm, 0.2 mm thickness) were cut from the film samples and fixed on the inner side of a spectrophotometer cell (with dimension of 1 cm), with further recording of the absorbance spectra. The determination was performed in triplicate.

## 3. Results and Discussion

### 3.1. Compatibilized Wood–Polymer Composites

In recent decades, studies on compatibilized thermoplastic polymer–clay–wood composites manufactured by melt mixing have emphasized the beneficial overall effect of clay and compatibilizer on the bulk properties of the material due to their synergy. The better the interfacial interactions, the greater the amount of wood waste that can be incorporated into composites. Suitable compatibilizers for polyolefins are widely available commercially (malleated HD- or LD-polyolefins, such as PE-*g*-MA or MAPE, PP-*g*-MA or MAPP) and have various degrees of grafting and different molecular weight. Their selection for a given multicomponent system must take into account the structural similarity with the matrix polymer in order to optimize compatibilization (e.g., PE and PP are incompatible polymers; therefore, the suitable compatibilizer is their malleated derivative). Regarding clay, it was confirmed that its addition in small amounts to the WPC formulation significantly improved not only the mechanical properties but also the fire retardancy [56,57,58].

This study aimed to assess the combined effects of a compatibilizer (MAPP) and an organically modified montmorillonite (Cloisite 20A) on the properties of some compatibilized wood–polypropylene composites. Investigation into thermal (TGA, DSC) and mechanical (stress–strain tests) characteristics allowed us to evaluate the evolution of the composite properties upon the addition of various amounts of wood at predetermined contents of MAPP and clay.

Composite formulations and their corresponding sample codes are presented in Table 1. The selected compatibilizing agent was the malleated PP (MAPP) due to its structural similarity with the matrix. As for the clay, Cloisite 20A (Cl 20A) was selected due to its structure: as an organically modified clay, it contains units of dimethyl bis(hydrogenated tallow) quaternary ammonium chloride, where the hydrogenated tallow is a blend of saturated alkyl chains, which also contributes to the overall compatibilization effect. Concerning the ratio of the components in composites, this parameter was set according to our previously presented conclusions [59,60,61,62,63] which refer to the fact that WPCs’ mechanical properties have higher values when loads of compatibilizer and clay are medium, while the amount of wood is medium to high. Furthermore, due to the increased amount of clay, the composites will have enhanced fire retardancy. This approach will prove very useful for recycling larger amounts of wood waste per unit volume of the polymer matrix.

#### 3.1.1. Granulometric Study of Wood Particles

**Figure 3:**
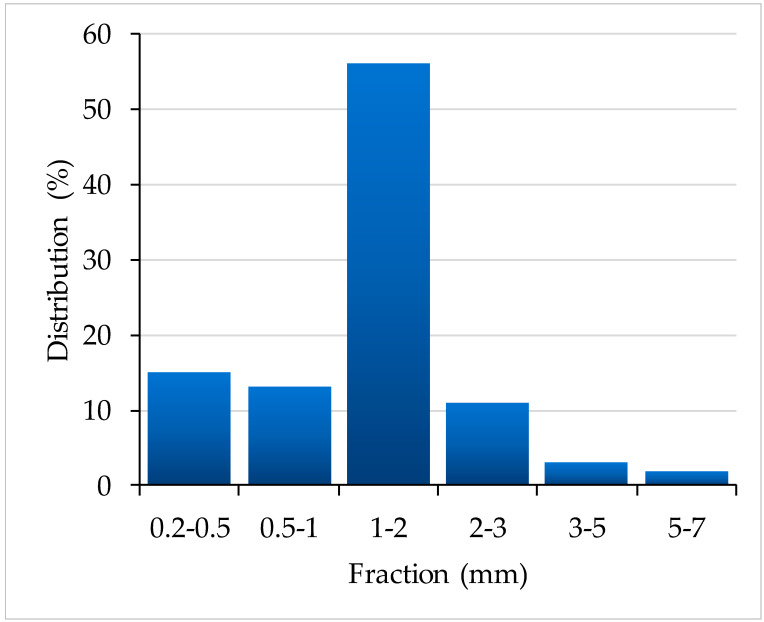
The granulometric distribution of wood particles.

Two different types of granulated fillers have been used to prepare these composites: Cloisite 20A, with a well-defined particle size distribution (<15μm, approx. 90%), and wood with particle sizes ranging between 0.2 and 7 mm. Therefore, it was necessary to identify the granulometric distribution of wood particles as this parameter strongly influences the interfacial interactions in composites and, subsequently, their mechanical properties. The particle size was determined as presented in Section 2.1.1. and each fraction was weighed. The granulometric distribution (percentage) of wood chips is presented in Figure 3.

Two related parameters have been defined for the wood particles as filler, according to reference [64]: the packing factor (F) and the density of the wood particles (*ρ_f_*). F was calculated for the statistical mixture of particles resulting from the granulometric study, namely by vibrational compacting of the wood chips in a glass measuring cylinder, and the value was F = 0.72. Theoretic calculations for wood particles of 1.00 and 0.2 mm in length gave close values F = 0.57 and 0.58, respectively. In this context, the value obtained for our mixture of wood particles is an indication that the distance between neighboring particles increased, thus allowing for a larger amount of polymer to cover (wet) the wood particles (forming a continuous phase) and even penetrate within through their tracheids. This will yield WPCs with improved elastic properties. As for the density of the wood particles, the calculated value for each fraction was *ρ_f_* = 0.487 g/cm^3^. Based on these data, it was concluded that it is possible to manufacture WPCs with a high wood content (50%) and very good mechanical properties fit for common applications.

#### 3.1.2. Thermal Behavior of Composites

**Figure 4:**
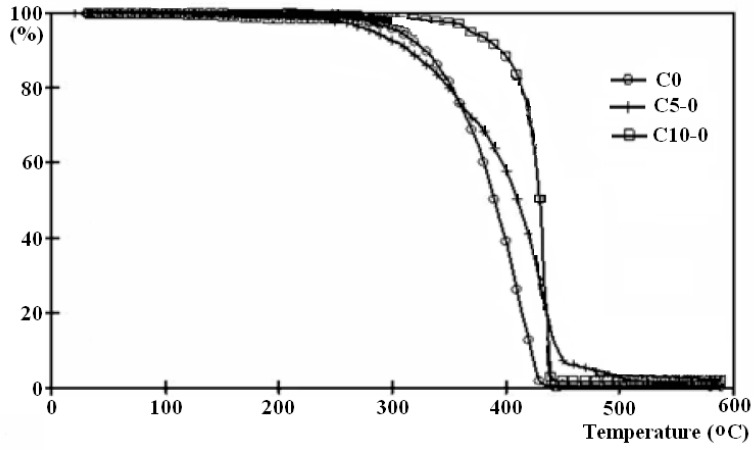
TGA thermographs of samples C0, C5-0, and C10-0.

TGA curves registered for samples without wood (C0, C5-0, and C10-0) indicated that upon the addition of clay and MAPP, the decomposition temperature increased at 50% weight loss as compared to raw PP due to two converging phenomena. On the one hand, there is the hindered diffusion of volatile decomposition products caused by the clay particles dispersed within the matrix, which created routes with pronounced tortuosity, thus increasing the barrier effect of clay [65]. On the other hand, the presence of MAPP favored the clay dispersion and hence improved barrier properties, which can also yield the physico-chemical adsorption of the volatiles onto the clay particles up to a certain degree [59,66]. TGA thermographs of samples C0, C5-0, and C10-0 are presented as examples in Figure 4.

DSC data for all samples (raw PP and composites) are presented in Table 3.

All composite samples had melting temperature values (*T_m_*) higher than that of neat PP. The highest value was registered for C10-30, a sample containing the highest amounts of compatibilizer and clay and a moderate amount of wood, while C10-50, the one with the highest amount of wood, had a lower *T_m_*. This trend was observed for all samples containing wood (15, 30, and 50%, respectively).

Samples with no wood (C0, C5-0, and C10-0) owe their behavior to the favorable effect of MAPP and clay present in composites. When low to moderate amounts of wood were added to the formulations, the matrix effectively wet the particles, forming a continuous phase that coated wood particles. Therefore, diffusion phenomena were slowed down, thus preventing thermal degradation. In addition, another effect must be considered: the cumulative contribution of clay and wood particles to the increased tortuosity of the path for volatiles. Furthermore, cellulose in wood is highly crystalline and grants wood thermal stability within certain limits. The balance of these phenomena is modified upon the further addition of wood (50%) as the polymer layer became thinner and, in effect, *T_m_* decreased correspondingly. 

The melting enthalpy (Δ*H_m_*) was determined at a heating rate of 10 °C/min. Experimental data indicated an increased thermal stability of samples along with the increasing amounts of clay and MAPP in the composite formulations. The addition of wood caused the decrease in the Δ*H_m_* values far below that of raw PP, most likely due to the fact that the wood particles, just like clay platelets, have absorbed more heat during the melting of the composites [67,68].

The crystallization temperature values (*T_c_*) increased upon the addition of MAPP and clay, and wood, correspondingly, which was a clear indication that the overall effect is the result of the synergistic action of the three elements: clay particles acted as nucleating agents, as expected, wood filler added to their effect, and MAPP modified the crystallinity by decreasing the nucleation density and the crystallization rate in composites [69].

On the other hand, the crystalline enthalpy (−Δ*H_m_*) showed a decreasing trend as MAPP, clay, and wood were added to the composite formulations. A possible conclusion of this behavior could be that the factors which are promoting the crystallization of PP, namely clay particles and wood chips, are, in fact, disrupting the crystallization in the composite bulk due, most likely, to the changes in samples rheology, interfacial interactions, and processes within the interphase between matrix and filler (both clay and wood). These findings are in perfect correlation with the experimental data for crystallinity (*X_c_*).

Thus, the higher the clay and wood content, the lower the crystallization rate and the higher the amount of heat absorbed. In fact, the higher amounts of clay and wood particles caused the increased thermal stability and, as a consequence, flame retardancy of composites provided that the wood particles remain covered with a polymer film of adequate thickness.

#### 3.1.3. Mechanical Properties

**Figure 5:**
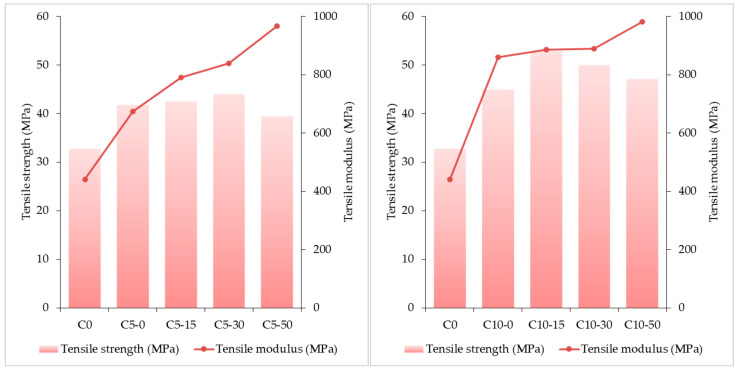
Mechanical properties of composites.

The tensile properties of PP and the corresponding composite samples are presented in Figure 5.

The variation of the tensile strength and modulus of composites compared with those of raw PP is closely related to the composition of samples. In the case of composites without wood (C0, C5-0, and C10-0), the values recorded for the tensile strength were a little higher than that of PP, but the values of the tensile modulus were significantly increased relative to the same reference. These findings seem to indicate that the intercalation of MAPP and intercalation/exfoliation of clay platelets inside the matrix (PP) during processing were highly effective, resulting in materials with increased tensile strength.

After adding wood chips to the formulations, the values of tensile modulus of most composites increased significantly, and the maximum value was recorded for sample C10-50. Thus, it may be concluded that the higher modulus is strongly related to the increased stiffness of composites due to the highest amount of wood filler.

As a trend, the addition of moderate amounts of MAPP and clay and low to moderate amounts of wood caused an increase in tensile strength. But its values decreased when the composites were loaded with the maximum amounts of wood. At the same time, the values of the tensile modulus increased. Therefore, it may be reasonably stated that high values of tensile strength confirmed the highly improved bonding of PP and wood particles due to the presence of MAPP and clay. The interactions that occurred inside composites, both physical and chemical, enhanced the interfacial adhesion. Thus, MAPP and clay (both platelets and exfoliated sheets) interpenetrated with the PP macromolecules, allowing the wood particles to disperse within the matrix to a high degree of homogeneity. In this regard, small and medium size particles are favored, and thus they will be able, most likely, to reach more bonding sites. On the one hand, there are the hydrogen bonds that have formed. On the other hand, under processing conditions, ester bridges are likely to occur as a result of interactions between maleic anhydride moieties of MAPP and reactive –OH functional groups in wood. As a consequence, the interfacial adhesion was enhanced, hence the improved load transfer and, subsequently, higher tensile values [70,71].

When maximum amounts of wood were loaded, a decrease in tensile strength was registered, despite the significant content in MAPP and clay. This behavior might be attributed to the formation of a rigid wood skeleton (continuous phase) within the matrix when the polymer phase imperfectly wets the filler particles. Therefore, the composites have acquired increased stiffness and brittleness.

In addition, the cavitation phenomenon cannot be neglected when it comes to the mechanical deformation of PP [72]. Studies showed the effects of cavitation, shearing, and plastic deformation could lead to material failure so that increasing the crystalline phase by various methods (annealing, blending, compatibilization) can cause increased yield stress and volume strain. At the same time, cavitation influences the toughness of the material [72,73,74]. The cavitation generally occurs around the yield point and consists of the formation of micro-/nanometric cavities in the bulk of the polymer. This phenomenon is rather dynamic, meaning that (1) the size and orientation of cavities change during the mechanical stress, and (2) new cavities are formed while others disappear. Increasing the testing temperature will limit the polymer’s ability to cavitate [75,76,77]. Taking into account these data, it is reasonable to assume that cavitation of PP under the processing conditions (190 °C; 100–150 rpm) affected the dispersion of particulate fillers (clay and wood) up to a certain degree, despite the beneficial effect of temperature and MAPP. The detachment of rigid particles from the matrix can occur up to a certain extent, locally, where cavitation is more pronounced, contributing to the overall effect of decreased tensile strength and enhanced tensile modulus.

Nevertheless, the complexity of processes that take place within these composites is far from being completely elucidated.

### 3.2. Wood–Plastic Composites with Starch Matrix

#### 3.2.1. FTIR Spectroscopy Investigation

**Figure 6:**
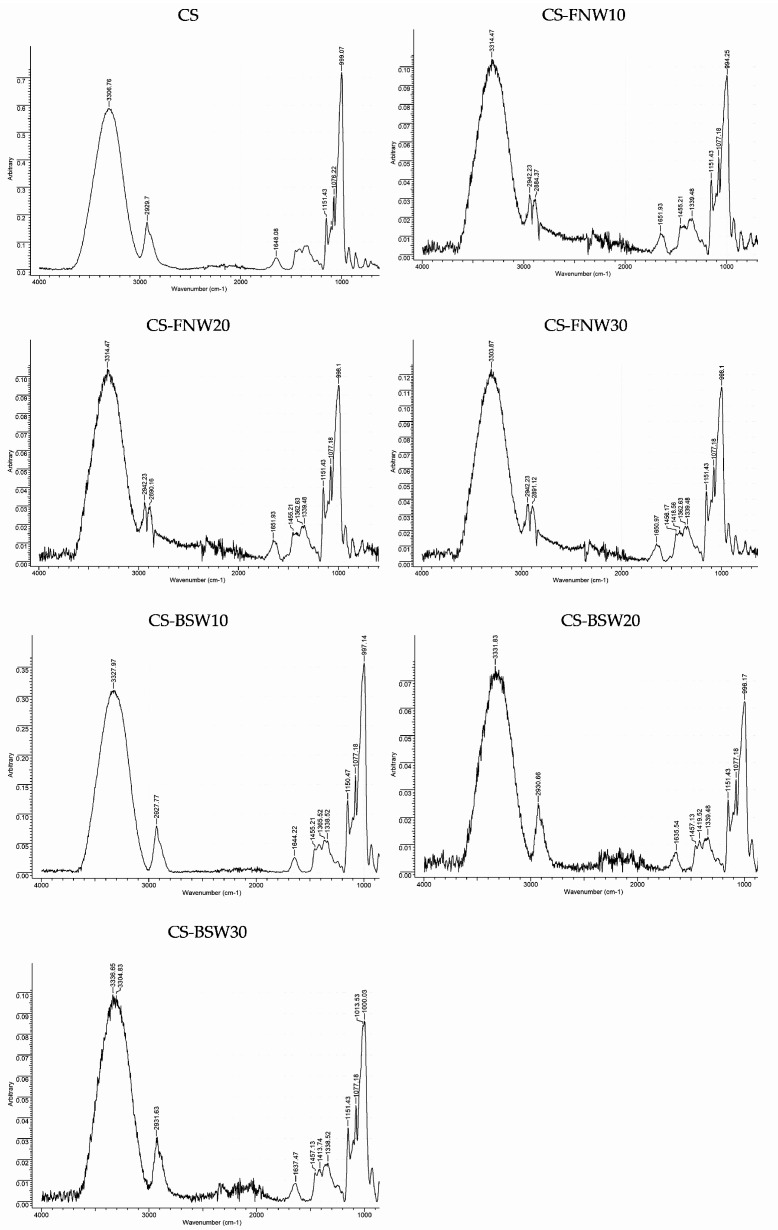
FTIR spectra for initial composite samples made from plasticized starch and for composite samples with lignocellulose waste fillers (fir tree needles, beech wood sawdust).

FTIR spectroscopy was employed in order to evidence the interactions between plasticized starch and lignocellulose waste (FNW, BSW) in composite films. The FTIR spectra are given in Figure 6.

The samples, both initial plasticized starch and those with wood waste in composition, have similar spectral characteristics in two band regions from the FTIR spectra, except the peaks at 1650 and 1017 cm^−1^, which are related to the absorption of the hydroxyl groups [78]. The absorption band noticed at 3330 cm^−1^ in all composite samples can be attributed to the mixed hydroxyl groups from polysaccharides components (cellulose and hemicelluloses) present in FNW and BSW. The absorption band observed at around 1076 cm^−1^ in FTIR spectra for composite formulations with BSW is specific to the stretching of C-O in polysaccharides, respectively, while bands noticed at around 1651 cm^−1^ (in FNW composites) and around 1640 cm^−1^ (in BSW composites) are absorption bands specific to the lignin component as conjugated carbonyl group C=O. The absorption bands observed at around 2930 cm^−1^ and 1456 cm^−1^ (in BSW composites) are the characteristic peaks of methylene groups, being attributed to the symmetric stretching, asymmetric stretching, and in-plane deformation of the C-H bond in polysaccharides components. The absorption band from 1077 cm^–1^ corresponds to the carbonyl (C=O) stretching and C-O stretching, mainly in polysaccharides from BSW [79].

#### 3.2.2. WAXD Analysis

**Figure 7:**
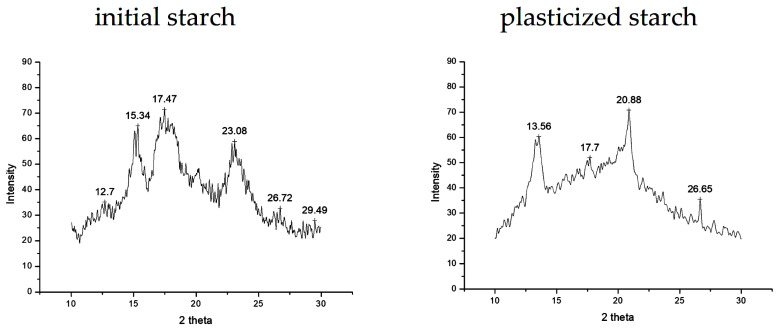
WAXD patterns recorded for initial corn starch and plasticized corn starch film.

The pattern recorded for corn starch, a semi-crystalline polysaccharide, evidences specific sharp diffraction peaks of A-type crystalline starch (glucose units joined by glycosidic bonds are packed densely) at 15.3°, 17.5°, and 23.1° (2θ), respectively—see Figure 7.

One can observe that the crystalline part of the starch is reduced in plasticized starch because, after plasticization, the glycerol and water molecules altered the hydrogen bonds into initial starch macromolecules (both at intermolecular and intramolecular levels).

#### 3.2.3. Moisture Absorption Measurements

**Figure 8:**
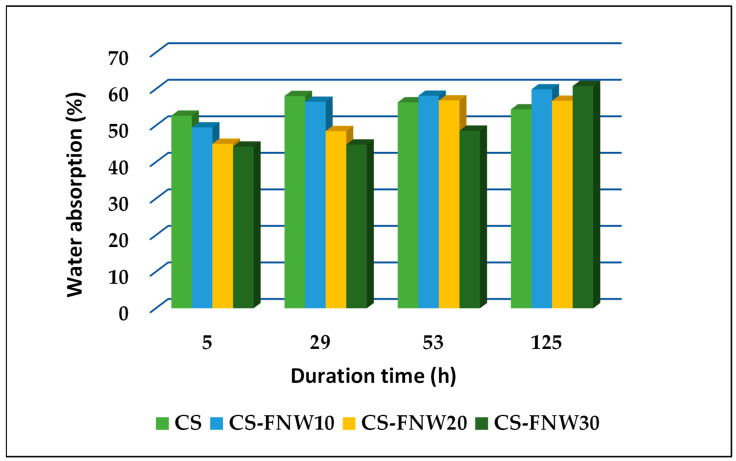
Water absorption evolution for plasticized starch composite samples with fir tree needles waste.

**Figure 9:**
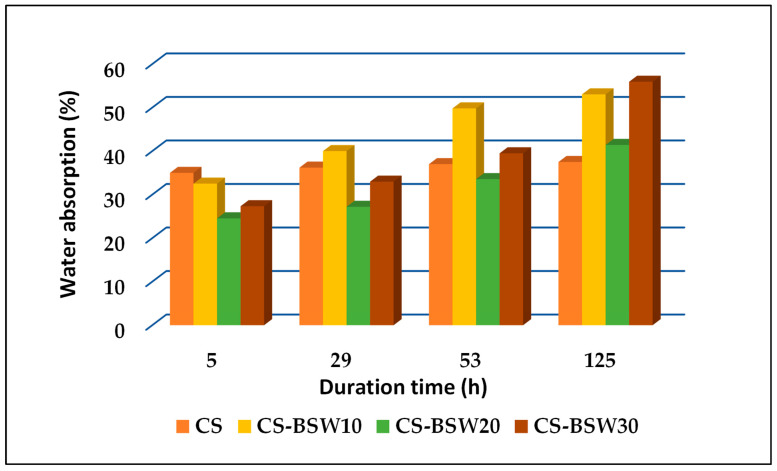
Water absorption evolution for plasticized starch composite samples with beech wood sawdust waste.

The moisture absorption investigation is important for understanding the performance of starch-based composites since the moisture retention under immersion in water or exposure to a high-humidity environment intimately relates to such composite properties as dimensional stability and mechanical strength. Though natural polymers-based materials (i.e., lignocellulose, holocellulose, cellulose, lignin, starch) are considered the most promising materials for producing biodegradable composites, their wide range of applications is limited due to their poor resistance towards moisture. The addition of appropriate fillers is an effective way of decreasing the sensitivity to moisture and improving mechanical properties. Moisture absorption values were evaluated for all composite film samples (see Figure 8 and Figure 9). These values decreased with the increasing amount of waste in composite formulations, a fact that denotes some resistance to the presence of moisture.

#### 3.2.4. Optical Properties Measurements

**Figure 10:**
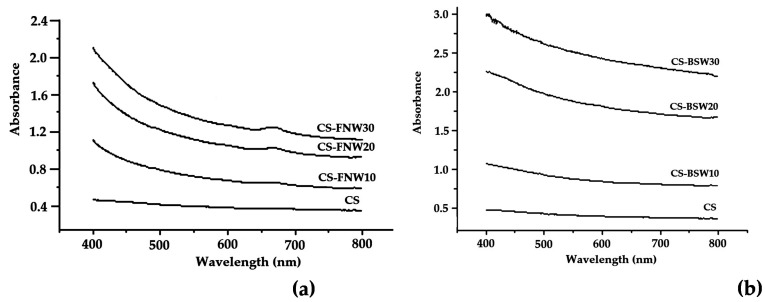
Optical properties (opacity values) for plasticized starch composite samples with wood waste: FNW (**a**) and BSW (**b**).

Optical properties are of real significance for starch composite films when applications such as food packaging or coating are envisaged. If particle sizes are larger than the visible wavelength, opaque composite materials can result, so it is very useful to investigate such properties. Figure 10a,b illustrates the influence of FNW and BSW addition on the transparency properties of starch-based composite films.

A decrease in transparency can be observed with the increase in FNW and BSW content of the films (see Figure 10), a tendency that is more noticed when BSW is used in the composite, probably due to its larger particle size. Interaction of light radiation with the materials’ surface implies phenomena such as reflection, absorption, or transmission with highlighting color, gloss, and transparency properties. In the case of multicomponent polymer films, the diffusion of light occurs mainly at the interface of the polymer components, impeding its further transmission. FNW and BSW particles are not water soluble and are randomly dispersed into the plasticized starch matrix during composites manufacturing (due to the water and glycerol), thus providing a larger interfacial area within the structure of the film. This further enhances the light diffusion and confers an attenuated transparency to the composite films. The reduced content of FNW and BSW in composite formulations has some beneficial effects on transparency in relation to particle size.

## 4. Conclusions

Feasible opportunities for producing WPCs with value-added properties starting from recycled materials, namely wood waste, and intended for common applications have been explored. Given the advances recorded in recent decades, the relevance, both theoretical and practical, of this field of research becomes obvious. Our study illustrated two of the most important directions to develop WPCs: the use of (1) synthetic polymers, namely PP and malleated PP, as compatibilizers, and (2) natural polymers, namely glycerol-plasticized starch, as a matrix. Different types of wood waste have been considered: pine wood sawdust, fir tree needles (FNW), and beech wood sawdust (BSW). A wise choice of materials (i.e., type of polymer matrix, employment of a compatibilizer and/or other additives, use of specific treatments for wood waste, etc.) and formulations for these multicomponent polymer systems yielded composites with complex supramolecular structures and improved bulk properties (e.g., enhanced durability—mechanical properties, water sorption—when used in outdoor applications). An efficient strategy to upgrade WPCs properties was to employ nanoparticles in their formulation as they can impart valuable characteristics, such as increased mechanical properties, flame retardancy, hydrophobicity, and resistance to photochemical degradation and biological attack. Thus, for WPCs made of PP, MAPP, Cloisite 20A, and wood, it has been demonstrated that it is possible to obtain composites with a high wood content and properties that make them suitable for common applications. The higher the clay and wood content, the lower the crystallization rate and the higher the amount of heat absorbed. In fact, the higher amounts of clay and wood particles caused the increased thermal stability and, as a consequence, flame retardancy of composites. At the same time, the tensile modulus of composites increased along with the content of clay and wood as a result of the enhanced stiffness, while the tensile strength slightly decreased. In the case of WPCs made of glycerol-plasticized starch as a matrix and moderate amounts of lignocellulosic waste, some other properties (water absorption, transparency) have been considered and discussed as being very important features when selecting the suitable material for a certain application. Experimental results indicated that values for water absorption decreased with the increasing amount of lignocellulose waste in composite formulations, which denotes some resistance of WPCs towards moisture. The reduced content of FNW and BSW in composite formulations had a beneficial effect on transparency, up to a certain extent, in relation to the particle size and light diffusion (multiple processes of reflection and refraction, absorption, and transmission of light).

In conclusion, this study successfully illustrated the two main directions of re- and up-cycling wood waste by manufacturing WPCs with polymer matrices of different natures and moderate to high content of reinforcement, as well as the possibility to adjust some WPCs’ properties by modulating their composition so that they are suitable for common application. However, the numerous and complex interactions that occur in the interphase formed in these materials and at the interface between different components are far from being fully elucidated.

## Figures and Tables

**Table 1:** Composites formulation and sample code for PP-based materials.

Sample Code	Matrix PP	Compatibilizer MAPP	Cloisite Cl20A	Wood Chips
C0	100	0	0	0
C5-0	100	5	5	0
C5-15	100	5	5	15
C5-30	100	5	5	30
C5-50	100	5	5	50
C10-0	100	10	10	0
C10-15	100	10	10	15
C10-30	100	10	10	30
C10-50	100	10	10	50

**Table 2:** Composites formulation and sample code for starch-based materials.

Sample Code	Composition
CS	plasticized corn starch film
CS-FNW10	plasticized corn starch film with 10% fir tree needles waste
CS-FNW20	plasticized corn starch film with 20% fir tree needles waste
CS-FNW30	plasticized corn starch film with 30% fir tree needles waste
CS-BSW10	plasticized corn starch film with 10% beech wood sawdust waste
CS-BSW20	plasticized corn starch film with 20% beech wood sawdust waste
CS-BSW30	plasticized corn starch film with 30% beech wood sawdust waste

**Table 3:** DSC data for PP and composite samples.

Sample Code	*T_m_* (°C)	Δ*H_m_* (J/g)	*T_c_* (°C)	−Δ*H_m_* (J/g)	*X_c_* (%)
**C0**	163.1	81.5	108.9	91.0	39.01
**C5-0**	169.5	90.2	113.1	88.9	44.20
**C5-15**	167.7	86.0	118.5	83.7	42.48
**C5-30**	168.1	79.4	118.0	75.5	35.77
**C5-50**	164.1	68.9	119.3	72.8	32.00
**C10-0**	168.5	92.0	114.7	89.7	44.69
**C10-15**	166.6	86.9	116.6	83.9	39.90
**C10-30**	168.9	78.1	118.9	79.4	35.17
**C10-50**	162.9	67.4	121.1	70.5	32.25

*T_m_* = melting point; Δ*H_m_* = melting enthalpy; *T_c_* = crystalline temperature; −Δ*H_m_* = crystalline enthalpy; *X_c_* = crystallinity.

## Data Availability

Not applicable.

## References

[1] Besserer A., Troilo S., Girods P., Rogaume Y., Brosse N. (2021). Cascading Recycling of Wood Waste: A Review. Polymers.

[2] Commission E. A New Circular Economy Action Plan for a Cleaner and More Competitive Europe. https://environment.ec.europa.eu/strategy/circular-economy-action-plan_en.

[3] Commission E. New Waste Rules Will Make EU Global Front-Runner in Waste Management and Recycling. https://commission.europa.eu/news/new-waste-rules-will-make-eu-global-front-runner-waste-management-and-recycling-2018-04-18_en.

[4] Mazzanti M., Zoboli R., D’Amato A., Mazzanti M., Montini A. (2013). Waste management in spatial environments. Waste Management in Spatial Environments.

[5] Bergeron F.C. (2014). Assessment of the coherence of the Swiss waste wood management. Resour. Conserv. Recycl..

[6] Olszewski A., Kosmela P., Piszczyk Ł. (2023). A novel approach in wood waste utilization for manufacturing of catalyst-free polyurethane-wood composites (PU-WC). Sustain. Mater. Technol..

[7] Basalp D., Tihminlioglu F., Sofuoglu S.C., Inal F., Sofuoglu A. (2020). Utilization of Municipal Plastic and Wood Waste in Industrial Manufacturing of Wood Plastic Composites. Waste Biomass Valorization.

[8] Diestel S., Krause A. (2018). Wood-based composites with thermoplastic polyurethane as matrix polymer. J. Appl. Polym. Sci..

[9] Bodîrlău R., Teaca C.A., Spiridon I. (2014). Green composites comprising thermoplastic corn starch and various cellulose-based fillers. BioResources.

[10] Kosakowski W., Bryszewska M.A., Dziugan P. (2020). Biochars from Post-Production Biomass and Waste from Wood Management: Analysis of Carbonization Products. Materials.

[11] Zhou H., Li W., Hao X., Zong G., Yi X., Xu J., Ou R., Wang Q. (2022). Recycling end-of-life WPC products into ultra-high-filled, high-performance wood fiber/polyethylene composites: A sustainable strategy for clean and cyclic processing in the WPC industry. J. Mater. Res. Technol..

[12] Tang W., Xu J., Fan Q., Li W., Zhou H., Liu T., Guo C., Ou R., Hao X., Wang Q. (2022). Rheological behavior and mechanical properties of ultra-high-filled wood fiber/polypropylene composites using waste wood sawdust and recycled polypropylene as raw materials. Constr. Build. Mater..

[13] Dong C., Parsons D., Davies I.J. (2014). Tensile strength of pine needles and their feasibility as reinforcement in composite materials. J. Mater. Sci..

[14] Thakur V.K., Singha A.S. (2011). Physicochemical and Mechanical Behavior of Cellulosic Pine Needle-Based Biocomposites. Int. J. Polym. Anal. Charact..

[15] Gupta M., Chauhan M., Khatoon N., Singh B. (2010). Composite boards from isocyanate bonded pine needles. J. Appl. Polym. Sci..

[16] Singha A.S., Thakur V.K. (2009). Study of mechanical properties of urea-formaldehyde thermosets reinforced by pine needle powder. BioResources.

[17] Nemli G., Yildiz S., Derya Gezer E. (2008). The potential for using the needle litter of Scotch pine (*Pinus sylvestris* L.) as a raw material for particleboard manufacturing. Bioresour. Technol..

[18] Hughes M., Baillie C. (2004). Applications. Green Composites: Polymer Composites and the Environment.

[19] Mehmood S., Khaliq A., Ranjha S.A. The use of post-consumer wood waste for the production of wood plastic composites: A review. Proceedings of the Third International Symposium on Energy from Biomass and Waste.

[20] Martinez Lopez Y., Paes J.B., Gustave D., Gonçalves F.G., Méndez F.C., Theodoro Nantet A.C. (2020). Production of wood-plastic composites using cedrela odorata sawdust waste and recycled thermoplastics mixture from post-consumer products—A sustainable approach for cleaner production in Cuba. J. Clean. Prod..

[21] Keskisaari A., Kärki T. (2018). The use of waste materials in wood-plastic composites and their impact on the profitability of the product. Resour. Conserv. Recycl..

[22] Ayrilmis N., Kaymakci A., Güleç T. (2015). Potential use of decayed wood in production of wood plastic composite. Ind. Crops Prod..

[23] Ge S.B., Gu H.P., Ma J.J., Yang H.Q., Jiang S.C., Liu Z., Peng W.X. (2018). Potential use of different kinds of carbon in production of decayed wood plastic composite. Arab. J. Chem..

[24] Hyvärinen M., Ronkanen M., Kärki T. (2019). The effect of the use of construction and demolition waste on the mechanical and moisture properties of a wood-plastic composite. Compos. Struct..

[25] Nukala S.G., Kong I., Kakarla A.B., Kong W., Kong W. (2022). Development of Wood Polymer Composites from Recycled Wood and Plastic Waste: Thermal and Mechanical Properties. J. Compos. Sci..

[26] Chun K.S., Subramaniam V., Yeng C.M., Meng P.M., Ratnam C.T., Yeow T.K., How C.K. (2018). Wood plastic composites made from post-used polystyrene foam and agricultural waste. J. Thermoplast. Compos. Mater..

[27] Mohammed A.S., Meincken M. (2021). Properties of Low-Cost WPCs Made from Alien Invasive Trees and rLDPE for Interior Use in Social Housing. Polymers.

[28] Bavasso I., Marzi D., Bracciale M.P., Di Palma L., Tirillò J., Sarasini F. (2022). Plant Waste as Green Reinforcement for Polymer Composites: A Case Study of Pteris Vittata Roots. J. Nat. Fibers.

[29] Bütün F.Y., Sauerbier P., Militz H., Mai C. (2019). The effect of fibreboard (MDF) disintegration technique on wood polymer composites (WPC) produced with recovered wood particles. Compos. Part A Appl. Sci. Manuf..

[30] Singh T., Lendvai L., Dogossy G., Fekete G. (2021). Physical, mechanical, and thermal properties of Dalbergia sissoo wood waste-filled poly(lactic acid) composites. Polym. Compos..

[31] Sommerhuber P.F., Welling J., Krause A. (2015). Substitution potentials of recycled HDPE and wood particles from post-consumer packaging waste in Wood–Plastic Composites. Waste Manag..

[32] Barbos J.D.V., Azevedo J.B., Cardoso P.d.S.M., da Costa Garcia Filho F., del Río T.G. (2020). Development and characterization of WPCs produced with high amount of wood residue. J. Mater. Res. Technol..

[33] Boonsri T., Rukchonlatee S., Jangchud I. (2022). Wood plastic composites (WPCs) from multilayer packaging wastes and rHDPE as pallets for green industry. IOP Conf. Ser. Mater. Sci. Eng..

[34] Tanasă F., Zănoagă M., Teacă C.A., Nechifor M., Shahzad A. (2020). Modified hemp fibers intended for fiber-reinforced polymer composites used in structural applications—A review. I. Methods of modification. Polym. Compos..

[35] Fang Y.G., Zhou Y.J., Lin J.Y., Lin Y.L., Li Z.H., Yang L.T., Yang C.L., Wang Z.Y. (2023). Influences of Polycarboxylic Acid EDTA on the Compatibility and Physical Properties of Sandal Wood Flour Reinforced Poly(lactic acid)/poly(butylene adipate-co-terephthalate) Biocomposites. J. Polym. Environ..

[36] Kord B., Ghalehno M.D., Movahedi F. (2020). Effect of immidazolium-based green solvents on the moisture absorption and thickness swelling behavior of wood flour/polyethylene composites. J. Thermoplast. Compos. Mater..

[37] Deka B.K., Dutta N., Maji T.K. (2011). Effect of Different Compatibilisers and Nanoclays on the Physical Properties of Wood (Phragmites Karka)–Polymer Composites. Polym. Renew. Resour..

[38] Martikka O., Kärki T. (2019). Promoting Recycling of Mixed Waste Polymers in Wood-Polymer Composites Using Compatibilizers. Recycling.

[39] Nagraik P., Shukla S.R., Kelkar B.U., Paul B.N. (2023). Wood modification with nanoparticles fortified polymeric resins for producing nano-wood composites: A review. J. Indian Acad. Wood Sci..

[40] Fufa S.M., Hovde P.J. Nano-based modifications of wood and their environmental impact: Review. Proceedings of the 11th World Conference of Timber Engineering.

[41] Rajan R., Näkki J., Layek R., Rainosalo E. (2021). Wood plastic composites with improved electrical and thermal conductivity. Wood Sci. Technol..

[42] Kumar S., Saha A. (2021). Graphene nanoplatelets/organic wood dust hybrid composites: Physical, mechanical and thermal characterization. Iran. Polym. J. (Eng. Ed.).

[43] Mandal M., Halim Z., Maji T.K. (2020). Mechanical, moisture absorption, biodegradation and physical properties of nanoclay-reinforced wood/plant oil composites. SN Appl. Sci..

[44] Ghorbani M., Biparva P., Hosseinzadeh S. (2018). Effect of colloidal silica nanoparticles extracted from agricultural waste on physical, mechanical and antifungal properties of wood polymer composite. Eur. J. Wood Wood Prod..

[45] Saieh S.E., Eslam H.K., Ghasemi E., Bazyar B., Rajabi M. (2019). Biodegradable composites of recycled thermoplastic starch and sawdust: The effect of cellulose nanofibers, nanoclay and temperature. Iran. Polym. J. (Eng. Ed.).

[46] Haque M.E., Khan M.W., Hasan M.M., Chowdhury M.N.K. (2022). Synthesis, characterization and performance of nanocopper impregnated sawdust-reinforced nanocomposite. Polym. Bull..

[47] Birinci E. (2023). Determination of technological properties of wood plastic nanocomposites produced by flat press reinforced with nano MgO. J. Compos. Mater..

[48] (2006). Environmental management — Life cycle assessment — Principles and framework.

[49] (2006). Environmental management — Life cycle assessment — Requirements and guidelines.

[50] Ramesh M., Rajeshkumar L., Sasikala G., Balaji D., Saravanakumar A., Bhuvaneswari V., Bhoopathi R. (2022). A Critical Review on Wood-Based Polymer Composites: Processing, Properties, and Prospects. Polymers.

[51] Sommerhuber P.F., Wenker J.L., Rüter S., Krause A. (2017). Life cycle assessment of wood-plastic composites: Analysing alternative materials and identifying an environmental sound end-of-life option. Resour. Conserv. Recycl..

[52] Khan M.M.H., Deviatkin I., Havukainen J., Horttanainen M. (2021). Environmental impacts of wooden, plastic, and wood-polymer composite pallet: A life cycle assessment approach. Int. J. Life Cycle Assess..

[53] Kaisersberger E., Mohler H. (1991). DSC on polymeric materials. NETZSCH Annu. Sci. Ind..

[54] Shafigullin L.N., Romanova N.V., Gumerov I.F., Gabrakhmanov A.T., Sarimov D.R. (2018). Thermal properties of polypropylene and polyethylene blends (PP/LDPE). Mater. Sci. Eng..

[55] (2021). Standard Test Method for Haze and Luminous Transmittance of Transparent Plastics.

[56] Lee Y.H., Kuboki T., Park C.B., Sain M., Kontopoulou M. (2010). The effects of clay dispersion on the mechanical, physical, and flame-retarding properties of wood fiber/polyethylene/clay nanocomposites. J. Appl. Polym. Sci..

[57] Kalali E.N., Zhang L., Shabestari M.E., Croyal J., Wang D.Y. (2019). Flame-retardant wood polymer composites (WPCs) as potential fire safe bio-based materials for building products: Preparation, flammability and mechanical properties. Fire Saf. J..

[58] Mokhena T.C., Sadiku E.R., Mochane M.J., Ray S.S. (2021). Mechanical properties of fire retardant wood-plastic composites: A review. Express Polym. Lett..

[59] Tanasă F., Zănoagă M., Nechifor M. (2014). Effect of an Organically Modified Nanoclay on the Properties of Some Compatibilized pp-Wood Composites. Rev. Roum. Chim..

[60] Teaca C.A., Tanasa F., Zanoaga M. (2018). Multi-component polymer systems comprising wood as bio-based component and thermoplastic polymer matrices—An overview. BioResources.

[61] Zanoaga M., Tanasa F., Mamunya Y. (2016). Compatibilized Green Composites Based on Wood Chips and Thermoplastic Polymer Waste Matrices. Cellul. Chem. Technol..

[62] Nechifor M., Tanasă F., Teacă C.A., Şulea D. (2022). Maleated coupling agents for the surface treatment of natural fibers. Surface Treatment Methods of Natural Fibres and Their Effects on Biocomposites.

[63] Tanasă F., Zănoagă M. (2018). Clay Reinforced Copmpatibilized Polyolefin Blends. Effect of the Interactions between Compatibilizer and Organically Modified Clay on Composites Properties. Rev. Roum. Chim..

[64] Mamunya Y., Zanoaga M., Myshak V., Tanasa F., Lebedev E., Grigoras C., Semynog V. (2006). Structure and properties of polymer–wood composites based on an aliphatic copolyamide and secondary polyethylenes. J. Appl. Polym. Sci..

[65] Qin H., Zhang S., Zhao C., Feng M., Yang M., Shu Z., Yang S. (2004). Thermal stability and flammability of polypropylene/montmorillonite composites. Polym. Degrad. Stab..

[66] Zanetti M., Camino G., Reichert P., Mülhaupt R. (2001). Thermal Behaviour of Poly(propylene) Layered Silicate Nanocomposites. Macromol. Rapid Commun..

[67] Kord B., Ravanfar P., Ayrilmis N. (2017). Influence of Organically Modified Nanoclay on Thermal and Combustion Properties of Bagasse Reinforced HDPE Nanocomposites. J. Polym. Environ..

[68] Lee S.Y., Kang I.A., Doh G.H., Kim W.J., Kim J.S., Yoon H.G., Wu Q. (2008). Thermal, mechanical and morphological properties of polypropylene/clay/wood flour nanocomposites. Express Polym. Lett..

[69] Arbelaiz A., Fernández G., Orue A. (2021). The effect of montmorillonite modification and the use of coupling agent on mechanical properties of polypropylene–clay nanocomposites. Polym. Polym. Compos..

[70] Lu J.Z., Negulescu I.I., Wu Q. (2012). Maleated wood-fiber/high-density-polyethylene composites: Coupling mechanisms and interfacial characterization. Compos. Interfaces.

[71] Spear M.J., Eder A., Carus M., Martin P.A. (2015). Wood polymer composites. Wood Composites.

[72] Pawlak A., Galeski A. (2010). Cavitation and morphological changes in polypropylene deformed at elevated temperatures. J. Polym. Sci. Part B Polym. Phys..

[73] Wang Y.X., Wu T., Fu Q. (2023). Competition of shearing and cavitation effects on the deformation behavior of isotactic polypropylene during stretching. Polymer.

[74] Na B., Lv R. (2007). Effect of cavitation on the plastic deformation and failure of isotactic polypropylene. J. Appl. Polym. Sci..

[75] Kida T., Hiejima Y., Nitta K.H., Yamaguchi M. (2022). Evaluation of microscopic structural changes during strain hardening of polyethylene solids using In situ Raman, SAXS, and WAXD measurements under step-cycle test. Polymer.

[76] Qian C., Zhao Y., Wang Y., Zhang C., Wang D. (2021). Tensile deformation mechanism of propylene-1-butene random copolymer: The role of initial crystalline morphology. Polymer.

[77] Pawlak A. (2013). Cavitation during tensile deformation of isothermally crystallized polypropylene and high-density polyethylene. Colloid Polym. Sci..

[78] Fang J.M., Fowler P.A., Tomkinson J., Hill C.A.S. (2002). The preparation and characterisation of a series of chemically modified potato starches. Carbohydr. Polym..

[79] Pandey K.K. (1999). A Study of Chemical Structure of Soft and Hardwood and Wood Polymers by FTIR Spectroscopy. J. Appl. Polym. Sci..

